# The impact of atrial mechanical function on age‐dependent presentation of neurocardiogenic syncope

**DOI:** 10.1002/clc.23704

**Published:** 2021-08-10

**Authors:** Melani Sotiriadou, Christodoulos E. Papadopoulos, Antonios P. Antoniadis, Panagiotis Roumelis, Stavros Vergopoulos, Periklis Konstantinidis, Efstathios D. Pagourelias, Stergios Tzikas, Nikolaos Fragakis, Vassilios Vassilikos

**Affiliations:** ^1^ Third Department of Cardiology, Hippokration General Hospital Aristotle University Medical School Thessaloniki Greece

**Keywords:** head‐up tilt test, left atrial strain, left atrial strain rate, left ventricular diastolic function, neurocardiogenic syncope, right atrial ejection fraction

## Abstract

**Background:**

The contribution of atrial and ventricular function in neurocardiogenic syncope (NCS) pathophysiology is elusive.

**Hypothesis:**

We assessed the influence of echocardiographic properties to the age of presentation and NCS recurrences.

**Methods:**

We assigned 124 patients with symptoms suggesting NCS, to those with syncope initiation at age <35 (group A, n = 56) and >35 years (group B, n = 68). Echocardiographic indices were measured before head‐up tilt test (HUTT).

**Results:**

A total of 55 had positive HUTT (44%) with a trend favoring group A (*p =* .08). Group A exhibited lower left atrial (LA) volume index (17 ± 6 vs. 22 ± 11 ml/m^2^, *p =* .015), higher LA ejection fraction (69 ± 10 vs. 63 ± 11%, *p =* .008), LA peak strain (reservoir phase 41 ± 13 vs. 31 ± 14%, *p =* .001, contraction phase 27 ± 11 vs. 15 ± 10%, *p <* .001) and LA peak strain rate (reservoir phase 1.83 ± 1.04 vs. 1.36 ± 0.96 1/s, *p =* .012, conduit phase 2.36 ± 1.25 vs. 1.36 ± 0.78 1/s, *p =* .001). Group A showed smaller minimum right atrial (RA) volume, better RA systolic function, superior left ventricular diastolic indices, and lower filling pressures. Group A patients were more likely to have >3 recurrences (82.0% vs. 50.1%, *p <* .05).

**Conclusions:**

Patients with younger age of NCS onset and more syncopal recurrences manifest smaller LA and RA dimensions with distinct patterns of systolic and diastolic function and better LA reservoir and contraction properties. These findings may indicate an increased susceptibility to preload reduction, thereby triggering the NCS mechanism.

## 1 INTRODUCTION

Neurocardiogenic syncope (NCS), is a common condition accounting for 50–66% of unexplained syncope in both children and adults.^1^, ^2^ Despite its high prevalence, the natural history and the underlying mechanisms of NCS are not well understood.^3^, ^4^, ^5^, ^6^ In addition, the reason that some people start fainting early with clusters of syncopal attacks while others later with long periods of syncope quiescence is also unknown.

Head‐up tilt test (HUTT) is frequently used as part of the diagnostic algorithm with a class IIa indication according to the latest European Society of Cardiology guidelines.^7^, ^8^, ^9^ The association however of HUTT outcome with anatomical and physiological cardiac chambers characteristics has not been sufficiently investigated especially using novel echocardiographic techniques like tissue doppler imaging (TDI) and speckle tracking echocardiography (STE).^10^, ^11^, ^12^ The systematic use of these modalities could contribute in the evaluation of the atrial and ventricular performance leading to further insights into the mechanism of NCS.^13^, ^14^, ^15^ The aim of this study, was to study the impact of anatomical and functional properties of cardiac chambers in the clinical presentation of syncope and the outcome of HUTT, in patients with NCS based on the age of syncope onset.

## 2 METHODS

**FIGURE 1:**
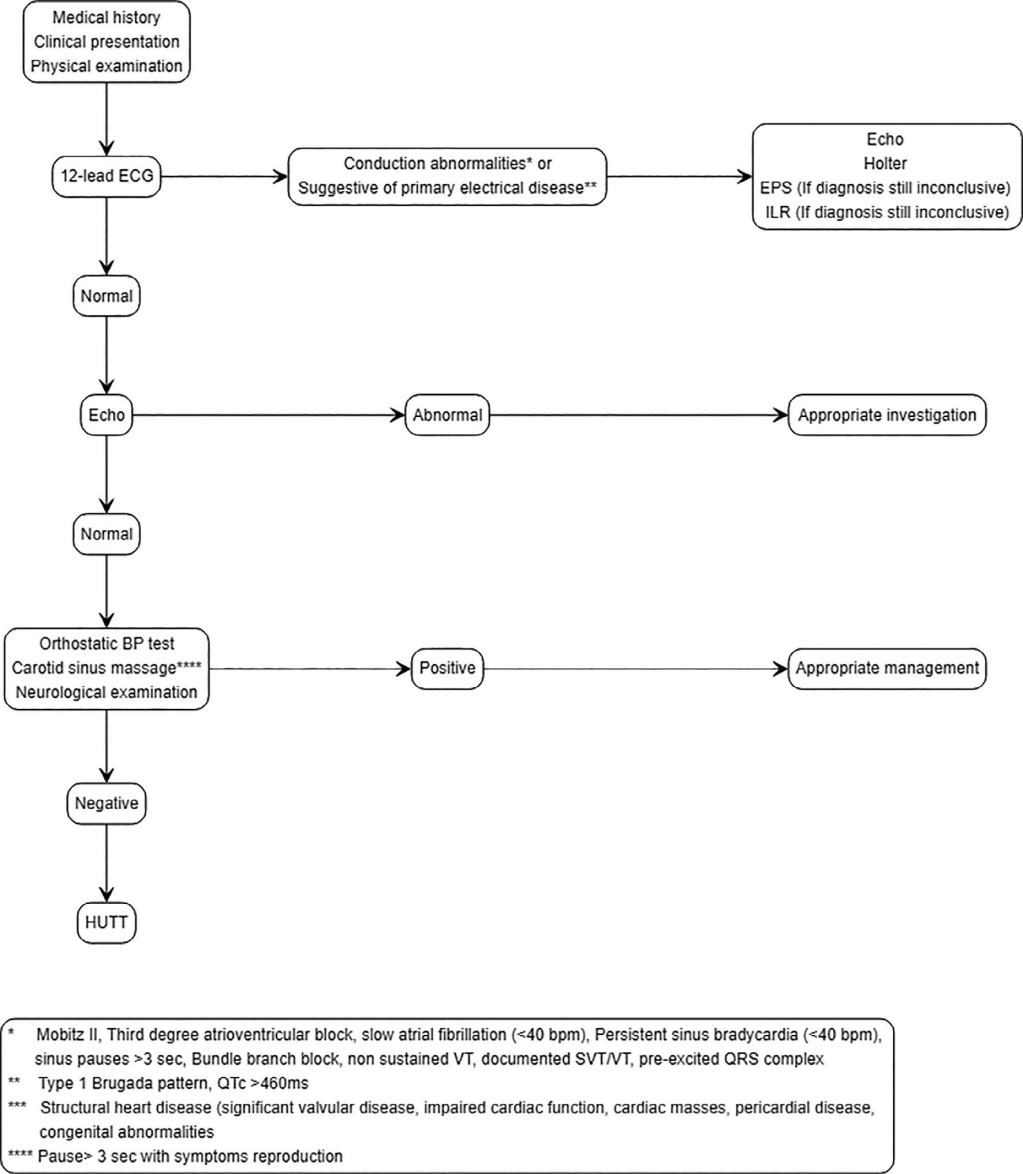
Stepwise patient evaluation. BP, blood pressure; ECHO, echocardiography; ECG, electrocardiogram; EPS, electrophysiological study; HUTT, head up tilt test; ILR, intravenous loop recorder

**FIGURE 2:**
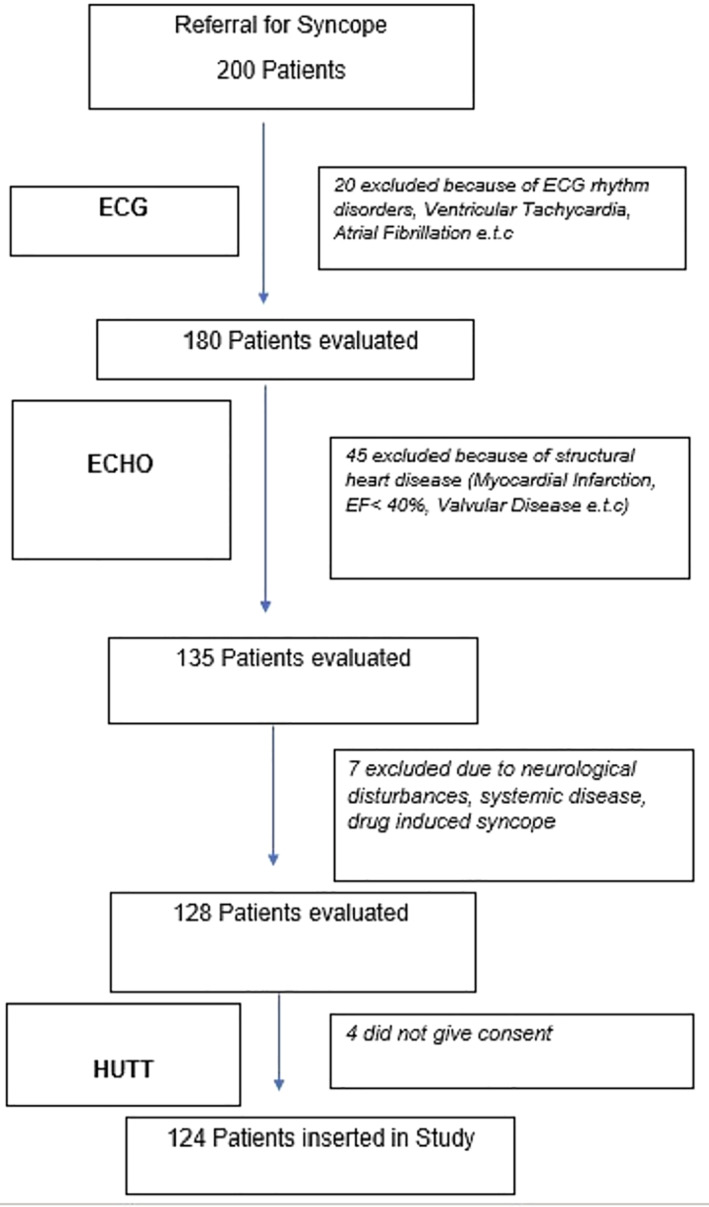
Patients' inclusion algorithm. ECHO, echocardiography; ECG, electrocardiogram; HUTT, head up tilt test

We studied 200 consecutive patients referred to our center for the investigation of syncope, presenting with symptoms suggestive of NCS. We excluded patients with underlying structural heart disease or conduction abnormalities that could be related to the mechanism of syncope. Patients with neurological or other diseases manifested with syncope were also excluded. Figure 1 illustrates our standard stepwise patient evaluation. Following this approach, 124 patients overall were included in the study (Figure 2).

We hypothesized that patients starting fainting at a younger age might have different clinical and echocardiographic features. We dichotomized therefore the patient population into two groups using as cut‐off the age of 35 based on previously published data.^16^, ^17^ All patients provided written informed consent. The study was approved by the Institutional Ethics Committee and was in accordance with the Declaration of Helsinki.

All patients underwent a comprehensive transthoracic echocardiography study 2–4 h before HUTT. All patients were in sinus rhythm at the time of the echocardiographic study. The basic left chambers anatomic and mechanical performance indices comprised left ventricular (LV) diameters and volumes, LV mass, LV ejection fraction (using Biplane Simpson's method), E and A wave velocity, E/A ratio, E wave deceleration time, E/e and left atrial (LA) volumes (minimal and maximal volumes at end‐diastole and end‐systole respectively). Additionally, the LA volumetric pump function was evaluated with the LA ejection fraction (LAEF). The basic right chambers anatomic and mechanical performance indices consisted of right ventricular (RV) basal diameter at end‐diastole, tricuspid annular plane systolic excursion (TAPSE), pulmonary artery systolic pressure (PASP), right atrial (RA) minimal and maximal volumes, RA ejection fraction (RAEF) and inferior vena cava dimensions. 2D STE was used to evaluate the LA mechanical deformation, by measuring the longitudinal LA strain and LA strain rate during three different phases: reservoir, conduit, and contraction phase, as previously described.^18^ All peak strain data were averaged and a single value for peak LA strain and peak LA strain rate were derived for each patient. Mean values (average of three measurements) for all measurements were used for the final analysis. All measurements were performed according to current guidelines and recent documentation in the literature.^18^, ^19^ Echocardiographic studies were performed using a commercially available ultrasound system (Vivid S5, GE Healthcare, Horton, Norway). Measurements including two‐dimensional (2D) STE were made offline using a commercially available ultrasound software package (EchoPAC GE, version 113, GE Healthcare, Milwaukee, WI). Two experienced blinded physicians (C. P. and M. S.) were responsible for the off‐line echocardiographic analyses. Inter‐ and intra‐observer variability for LA strain and LA strain rate measurements was performed in a group of 15 randomly selected patients and assessed using intra‐class correlation coefficient (two‐way mixed effects model).

HUTT was performed in a dark and quiet room by specialized personnel following a standard protocol.^4^, ^9^ A positive response was defined as the occurrence of syncope, accompanied by hypotension, bradycardia, or both and resembling the patient's original symptoms. All patients abstained from food and water for at least 4 h before HUTT. Medications that might alter the results of the examination were discontinued the day before. Each patient was placed in the supine position on the examination table after an intravenous catheter had been inserted into a peripheral upper limb vein at least 1 h before the procedure. Following a 10‐min resting phase, each patient was tilted upright at a 70° angle position for 20 min. If there was a positive response the patient was returned to the supine position and the test was terminated. If the response remained negative, Isoprenaline infusion was started at a dose of 1 μg/min titrated to elicit a 125%–130% increase of the heart rate. After titration, the patient remained in the tilting position for an additional 20 min or until developing a positive response. During the test, ECG and blood pressure were recorded noninvasively at 5‐min intervals or whenever the patient developed symptoms.

Continuous variables are expressed as mean ± SD, while the categorical ones as absolute values and percentages. The normality of distribution of the variables was checked by the Kolmogorov–Smirnov and Shapiro–Wilk tests. The comparison between normal variables was performed with the Student's *t*‐test while in the case of non‐normally distributed variables the Mann–Whitney U test was used. Possible correlations between the variables were investigated by calculating Pearson's r correlation coefficient in the case of normally distributed variables, and Spearman's rho correlation coefficient, in the case of non‐normally distributed variables. The predictive value of the variables in the occurrence of the study endpoints was investigated by accounting regression models. The level of statistical significance is set to 0.05. Statistical analyses were performed with SPSS (SPSS Inc., Chicago, IL), GraphPad Prism trial (GraphPad Software Inc., San Diego, CA), and Microsoft Excel Graphs (Office 365).

## 3 RESULTS

The study population comprised of 124 patients (mean age 47 ± 21 years, 71 female) with NCS divided into two groups. Group A consisted of patients with early syncope presentation (before the age of 35 years) and Group B of patients with late syncope presentation (after the age of 35 years). Table 1 summarizes the main characteristics of the study population.

**TABLE 1:** Study population characteristics

	Study population (n = 124)	Group A (n = 56)	Group B (n = 68)	*p‐*value
Age	47 ± 21	31 ± 16	59 ± 15	<.001
Male/female	53/71	17/39	36/32	<.05
Risk factors				
Hypertension (n, %)	30 (24)	5 (9)	25 (37)	<.001
Hyperlipidemia (n, %)	26 (21)	3 (5)	23 (34)	<.001
Diabetes mellitus (n, %)	2 (2)	0 (0)	2 (3)	NS
Renal disease (n, %)	3 (2)	0 (0)	3 (4)	NS
Smoking/alcohol (n, %)	19 (15)	8 (14)	11 (16)	NS
Comorbidities				
COPD (n, %)	4 (3)	1 (2)	3 (4)	NS
Stroke (n, %)	3 (2)	1 (2)	2 (3)	NS
Medications				
Beta‐blockers (n, %)	11 (9)	3 (5)	8 (12)	NS
ACEI/ARB (n, %)	7 (6)	1 (2)	6 (9)	NS
CCA (n, %)	1 (1)	0 (0)	1 (2)	NS
Diuretics (n, %)	1 (1)	0 (0)	1 (2)	NS
Combination of antihypertensives (n, %)	12 (10)	3 (5)	9 (13)	NS
No medications (n, %)	62 (50)	20 (36)	42 (62)	<.001
Diagnostic algorithm				
HUTT (N/P)	63/55	23/29	40/26	.08
Carotid massage (N/P)	111/2	50/0	61/2	NS
EPS (n, %)	10 (8)	2 (4)	8 (12)	NS
ILR (n, %)	4 (6)	0 (0)	4 (3)	NS

Abbreviations: ACEI, angiotensin converting enzyme inhibitor; ARB, angiotensin receptor blocker; CCA, calcium channel antagonist; COPD, chronic obstructive pulmonary disease; EPS, electrophysiology study; HUTT, head up tilt test; ILR, implantable loop recorder; N, negative; P, positive.

A positive response to HUTT was observed in 55 patients, a negative response to 63, while 6 patients exhibited a response consistent with postural orthostatic tachycardia syndrome (POTS). Two patients with positive carotid sinus massage which did not reproduce symptoms as well as 14 patients with non‐diagnostic ILR and EPS underwent further investigation with HUTT. A positive result in the passive phase was observed in 28, while 27 exhibited a positive result following Isoprenaline administration. A positive result was classified as mixed in 22, vasodepressive in 19, and cardio‐inhibitory in 14 patients. There was a trend for a positive HUTT in group A compared to group B patients (56% vs. 39%, *p =* .08).

Table 2 summarizes the different echocardiographic indices in Groups A and B, Group A patients showed smaller LV size compared to Group B as reflected by lower LV end‐systolic diameter (27 ± 4 vs. 29 ± 4 mm, *p =* .012) and less LV hypertrophy as indicated by lower interventricular septal diameter (8 ± 2 vs. 10 ± 2 mm, *p <* .001) and lower LV mass index (62 ± 30 vs. 93 ± 29 g/m^2^, *p <* .001). LV systolic function was superior in group A compared to group B as evidenced by LVEF (67 ± 5 vs. 64 ± 7%, *p =* .028). LV diastolic function was more favorable in Group A, as reflected by a higher E‐wave velocity, E/A ratio values, and a lower TDI E'‐wave mitral annulus velocity. Furthermore, LV diastolic filling pressures as reflected by E/E' were lower in Group A (5.8 ± 2.1 vs. 7.5 ± 3.5, *p <* .001).

**TABLE 2:** Echocardiographic parameters of the study population

	Group A	Group B	*p*‐value
LV indices			
LVEDD (mm)	42 ± 6	42 ± 6	NS
LVESD (mm)	27 ± 4	29 ± 4	.012
IVSd (mm)	8 ± 2	10 ± 2	<.001
LV mass index (g/m^2^)	62 ± 30	93 ± 29	<.001
LVEDV (ml)	70 ± 21	74 ± 27	NS
LVESV (ml)	24 ± 9	27 ± 12	NS
LVEF (%)	67 ± 5	64 ± 7	.028
LV GLS (%)	−21.8 ± 5.1	−19.0 ± 2.7	NS
E (m/s)	0.89 ± 0.23	0.69 ± 0.24	<.001
E/A	1.54 ± 0.62	0.99 ± 0.50	<.001
DT (ms)	212 ± 64	223 ± 59	NS
E' (m/s)	0.15 ± 0.04	0.10 ± 0.03	<.001
E/E'	5.8 ± 2.1	7.5 ± 3.5	.014
LA indices			
LA Vol max (ml)	30 ± 12	42 ± 21	.002
LA Vol index (ml/m^2^)	17 ± 6	22 ± 11	.015
LAEF (%)	69 ± 10	63 ± 11	.008
LA strain (%)			
Reservoir phase	41 ± 13	31 ± 14	.001
Conduit phase	14 ± 8	16 ± 10	NS
Contraction phase	27 ± 11	15 ± 10	<.001
LA strain rate (1/s)			
Reservoir phase	1.83 ± 1.04	1.36 ± 0.96	.012
Conduit phase	2.36 ± 1.25	1.36 ± 0.78	.001
Contraction phase	1.92 ± 0.83	1.77 ± 1.04	NS
RV indices			
RV base (mm)	30 ± 6	31 ± 7	NS
TAPSE (cm)	2.27 ± 0.28	2.22 ± 0.42	NS
PASP (mmHg)	19.5 ± 5.6	19.7 ± 7.6	NS
RA indices			
RA Vol max (ml)	27 ± 12	31 ± 16	NS
RA Vol max (ml)	11 ± 6	15 ± 11	.043
RAEF (%)	61 ± 11	54 ± 15	.024
IVC (mm)	13 ± 4	15 ± 4	NS

Abbreviations: E, early transmitral flow velocity; E', early diastolic mitral annular tissue velocity; E/A, E/A transmitral flow velocity ratio; IVC, Inferior vena cava diameter; IVSD, Intraventricular septal end diastolic diameter; LAEF, left atrial ejection fraction; LA SR, left atrial strain rate; LA strain, left atrial strain; LA Vol, left atrial volume (max, index) LVEDD, left ventricular end diastolic diameter; LVEDV, left ventricular end diastolic volume; LVEF, left ventricular ejection fraction; LVESD, left ventricular end systolic diameter; LVESV, left ventricular end systolic volume; LV GLS, left ventricular global longitudinal strain; LV Mass Index, left ventricular mass index; PASP, pulmonary artery systolic pressure; RAEF, right atrial ejection fraction; RA Vol, right atrial volume (min, max); RV Base, right ventricular basal diameter; TAPSE, tricuspid annular plane systolic excursion.

**FIGURE 3:**
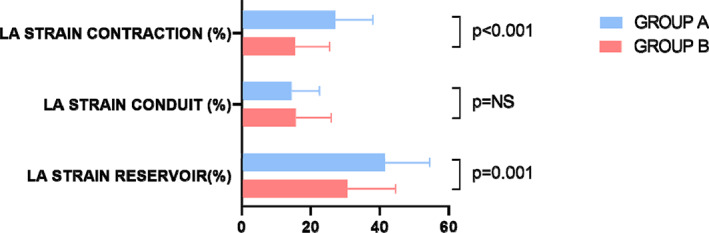
Left atrial strain values in Groups A and B

LA volume index was lower (17 ± 6 vs. 22 ± 11 ml/m^2^, *p =* .015) and LA ejection fraction was higher (69 ± 10 vs. 63 ± 11%, *p =* .008) in Group A patients. LA mechanical function evaluated by 2D STE was significantly better in Group A. LA peak strain during the reservoir phase and the contraction phase were higher in Group A (41 ± 13 vs. 31 ± 14%, *p =* .001 and 27 ± 11 vs. 15 ± 10%, *p <* .001 respectively), while LA peak strain rate during the reservoir phase and conduit phase (1.83 ± 1.04 vs. 1.36 ± 0.96 1/s, *p =* .012 and 2.36 ± 1.25 vs. 1.36 ± 0.78 1/s, *p =* .001 respectively) were higher as well, documenting a more pronounced LA mechanical function (Figure 3). Inter‐observer and intra‐observer variability for LA strain and LA strain rate measurements are as follows: Intra‐class correlation for inter‐ observer variability 0.971 (95% CI 0.873–0.995) for LA strain and 0.890 (95% CI 0.661–0.978) for LA strain rate, intra‐ observer variability for LA strain is 0.858 (95% CI 0.493–0.955) and for LA strain rate 0.890 (95% CI 0.761–0.968).

In terms of right heart, there were no significant differences between the two groups concerning RV dimensions and function. Group A patients showed smaller minimum RA volumes (11 ± 6 vs. 15 ± 11 ml, *p =* .043) and better RA systolic function (RA ejection fraction 61 ± 11 vs. 54 ± 15%, *p =* .024).When the study population was divided into two groups according to the number of recurrent syncopal episodes (<3 vs. >3), we found that 82.0% of patients in Group A exhibited more recurrences (>3) vs. 50.1% in Group B (odds ratio [OR] 3.6, 95% confidence Interval [CI] 1.47 to 8.23, *p <* .05), documenting that syncope patients with an early onset seem to be more susceptible to syncope recurrences.

### 3.1 Patient consent statement

Informed consent was obtained from all subjects involved in the study.

## 4 DISCUSSION

The main finding of this study is that patients who first experience episodes of NCS at a younger age demonstrate better LA and RA contractile function along with enhanced LV diastolic function compared to the subjects who commenced having NCS episodes at an older age.

Most people with NCS faint first in adolescence or early adulthood.^20^, ^21^ We speculated that fainters in younger ages may have different characteristics compared to older patients and this may have implications in their management. The POST study showed a different response of older vasovagal syncope patients to b‐blockade therapy compared to the younger suggesting a different underlying syncope mechanism. We tested this hypothesis in two prespecified and based on age 35 groups of patients according to the results derived from the Syncope Symptom Study and the POST study.^16^, ^17^

The LA plays an essential role in the filling of the LV and overall cardiac performance through its reservoir, conduit, and contractile function. The LA ejection fraction estimated with 2D echo was found superior in group A patients as well as the LA mechanical function evaluated by 2D STE. Two‐dimensional echocardiography gives an accurate estimation of the LA function, however the LA strain provides added value in the assessment of LA function and the estimation of LV filling pressure since it is less preload‐dependent than LA volume.^22^ Compared to the reported normal reference values for LA strain during the reservoir, conduit, and contraction phase (39.4%, 23%, and 17.4% respectively),^23^ our Group A patients had 41%, 14%, and 27% and Group B patients 31%, 16%, and 15% respectively. This finding might well indicate a super‐normal performance of LA during contraction in Group A patients and the significant contribution of this phase in LV filling under normal loading conditions.

It is acceptable that, during the initial phase of NCS an increased sympathetic activity with a surge of catecholamines (Epinephrine and Norepinephrine) takes place. There is evidence that a marked shift towards a greater circulation of epinephrine in younger compared to older patients with HUTT positive results occurs.^24^, ^25^ Because the b‐adrenergic properties of Epinephrine cause vasodilation and fluid volume shift in the splanchnic veins, it is expected to make subjects more susceptible to hypotension as a result of greater preload reduction.^26^ This could play a critical role in patients where the contributing role of LA function to the LV filling is stronger as occurs in Group A subjects. In contrast, older tilt‐positive patients exhibit lesser Epinephrine surge before syncope with reduced Epinephrine/Norepinephrine ratio maintaining a systemic vascular resistance above baseline levels.^24^ This suggests a syncopal mechanism less dependent on preload reduction and therefore LA function. Furthermore, this might explain the trend for a positive HUTT in Group A compared to Group B patients.

Although the prevailing theory of NCS supports an increase of ventricular contractility leading to activation of the cardiac mechanoreceptors several other reports suggest the scenario of a paradoxical decrease in myocardial contractility in patients with vasovagal syncope.^27^, ^28^ Liu et al, reported decreases in end‐systolic stress, cardiac index, and LV end‐diastolic volume as predictive of a positive tilt test.^15^ Goel et al, also reported a paradoxical decrease of LV strain in adults with NCS and a positive HUTT.^29^ The atria, which are rich in vagal innervation, could perform inadequately during vagal activation leading to a decrease in cardiac output. This pattern of response may have a more pronounced hemodynamic effect in Group A patients who demonstrate a super‐normal LA contraction suggesting a critical contribution of this specific phase in LV diastolic filling. This is in accordance with the findings by Moon et al, who described decreased left atrial volumes in HUTT positive patients and postulated a limited intracardiac volume reserve to play an important role in the mechanism of NCS.^30^ Although we found only a trend towards a positive HUTT in Group A patients, our results (small LA and LV) are perfectly in line with Moon et al findings.^30^

The function of the right cardiac chambers in vasovagal syncope is inadequately studied.^14^ We found that Group A patients have smaller minimum RA volumes and better RA systolic function compared to Group B patients suggesting a greater impact of right chambers to LV preload and cardiac output. When preload reduction is accentuated as occurs in Group A patients due to excessive splanchnic blood pooling, the contribution of RA to LV preload is further diminished, which may be critical in conditions of hypovolemic stress.^30^

In our study, early diastolic mitral velocity and E/A ratio were higher in Group A patients and were associated with a lower TDI E' wave mitral annulus velocity, reflecting a “healthier” LV diastolic function. The higher hypertrophy detected in Group B patients may account for this difference although it was within the normal range.

The clinical implications of this study relate to the observation that patients who experience syncopal events at early ages appear to have a better mechanical function of both atria and may express a higher contribution of these chambers to the LV preload. This means that any significant decrease in preload in conjunction with a reduction of the atrial mechanical performance when the vagal nerve predominates may have a serious adverse impact on cardiac output resulting in syncopal events. Therefore, Group A patients are likely to benefit more from hydration therapies, such as increased oral fluid intake and/or mineralocorticoid therapy to increase fluid retention.

## 5 STUDY LIMITATIONS

Our study has several limitations. Echocardiographic studies were not performed at the time of HUTT. Therefore, the hemodynamic changes are only speculated and were not proved. However, echocardiography was performed under normal loading conditions and stable hemodynamic status. Furthermore, intervendor variability regarding different ultrasound stations and different software strain analysis suites has to be taken into consideration. Although recent publications have reported standardization of the technique as well as normal ranges of LA strain, considerable intervendor variability seems to exist and steps must be taken to underscore this limitation.^31^ In this specific study, we used EchoPac software for LA strain analysis and our results should not be generalized and should be evaluated with caution.

The use of age 35 as a cutoff for further categorization of the study population into two groups was somewhat arbitrary. We prespecified this because it was the dichotomous diagnostic variable for vasovagal syncope that was derived from the Syncope Symptom Study.^32^ Furthermore, there was a difference in sex predominance between the two groups that might have influenced our results. Women were overrepresented in our population a finding that is frequent in syncope trials. However, this reflects the common incidence of vasovagal syncope in women, especially in high‐risk populations.

## 6 CONCLUSIONS

Conclusively, patients with early onset of NCS are more prone to syncopal recurrences. These patients exhibit better LA and RA mechanical function as well as “healthier” LV diastolic function. The above findings may indicate an increased susceptibility of this group to preload reduction, thereby triggering the mechanism of NCS. This subgroup of patients might respond favorably to more aggressive hydration management.

## CONFLICT OF INTEREST

The authors declare no conflicts of interest.

## ETHICS STATEMENT

The study was conducted according to the guidelines of the Declaration of Helsinki and approved by 1. Scientific Committee of Hippokration General Hospital of Thessaloniki (22.118, January 17, 2018) Ethics Committee of Aristotle University of Thessaloniki (3.292, May 2, 2018).

## Data Availability

The great majority of data are presented within this article, tables and figures. More data supporting reported results may be available on request from the corresponding author via databases from the Third Department of Cardiology.
